# Supplementary material for: A pilot acceptability evaluation of MomMind: A digital health intervention for Peripartum Depression prevention and management focused on health disparities
Source: PLOS Digit Health. 2024 May 22;3(5):e0000508. doi: 10.1371/journal.pdig.0000508 (PMC11111021; doi:10.1371/journal.pdig.0000508)
Supplement: S1 Appendix — (DOCX) [file pdig.0000508.s001.docx]

**S1 Appendix: “PPD 101” Scripts**

PPD 101: What is Perinatal Depression (PPD)?

| Cindy: This last month, I have been feeling very overwhelmed with the baby, and even sad sometimes. | 4.2 Information about antecedents |
| --- | --- |
| Doctor: It looks like you need help coping with being a new mom. |  |
| Cindy: Is this what people call the baby blues? |  |
| Doctor: The baby blues usually go away on their own in about two weeks. I think this can possibly be postpartum depression. It is more serious, but very common. It can happen for many reasons, like stressful events or hormonal changes. The good news is that there is a lot of help we can give you to cope better, through therapy or medication or both. | BCT 5.1 Information about health consequences |
| Cindy: But what if I can’t afford therapy? |  |
| Doctor: Don’t worry about that! We have an excellent social worker who can point you to any resources you may need. The important thing is that you have asked for help and are on your way to feeling better! | BCT 3.2 Social Support (practical and emotional) |

References and Evidence-based resources:

National Institute of Mental Health. (n.d.). Perinatal Depression. Accessed from: <https://www.nimh.nih.gov/health/publications/perinatal-depression>.

Centers for Disease Control and Prevention. (2022).  Depression During and After Pregnancy. Accessed from:

<https://www.cdc.gov/reproductivehealth/features/maternal-depression/index.html>

Postpartum Support International. (2022). Accessed from: <https://www.postpartum.net/>

PPD 101: Sleep and PPD

| Nurse: “Thank you for calling us! How can I help?”  Cindy: “Hi! I am having a lot of trouble sleeping with my newborn, and I feel exhausted”  Nurse: “Are you able to sleep when your baby sleeps?”  Cindy: “No, I always end up checking on him. Then the next day I feel in a very bad mood and sometimes even sad”. | 6.1 Demonstration of behavior  4.2 Information about antecedents |
| --- | --- |
| Nurse: “It *is* normal for a new mom to get a bit less sleep than normal. But if this becomes constant, then you made the right decision to call. It is important that you get at least a couple of hours of deep sleep every night.” | 9.1. Credible source |
| Cindy: “Is there anything I can do to sleep better?” |  |
| Nurse: “Yes:   - Take some time to unwind before bed. - Avoid screens. - Ask family for help with nighttime feedings. - Keep a sleep diary. | 3.2. Social support (practical)  4.1. Instruction on how to perform the behavior |
| Cindy: “Thank you!” |  |

References:

Sleep Foundation. (2022). “Postpartum Insomnia”. Accessed from: <https://www.sleepfoundation.org/insomnia/postpartum-insomnia>

Other Evidence-based resources.

Kimberly E. Monday, MD.

UT Health Neurosciences

(832) 325-7080

6410 Fannin Street, Suite 1014

Houston, TX 77030

Sarah Aguilar, FNP.

Houston Sleep and Headache Solutions.

13114 FM 1960 West, Ste 105B Houston, TX 77065

832-688-8886

PPD 101: Nutrition and PPD

| Doctor: How are you feeling with the morning sickness, Helen? |  |
| --- | --- |
| Helen: Well, I have been very sensitive to some smells. And, not being able to eat comfortably makes me a bit moody. | 4.2 Information about antecedents |
| Doctor: It looks like you have a very mild case of morning sickness. Here is what I recommend you can do:   - Make sure you stay hydrated. - Foods like ginger can help you fight off the morning sickness. - Eat snacks when you get hungry. This will help you stay in a better mood. | 6.1 Demonstration of behavior  9.1. Credible source |
| Helen: Sometimes I worry that this might affect the baby’s growth. |  |
| Doctor: As long as you are taking your prenatal vitamins and eating plenty of vegetables, fruits, protein, and dairy, both you and the baby should be excellent! | BCT 3.2 Social Support (practical and emotional) |
| Helen: Thank you! |  |

References and Evidence-based resources:

National Health Services. (2021). Vomiting and morning sickness. Accessed from: https://www.nhs.uk/pregnancy/related-conditions/common-symptoms/vomiting-and-morning-sickness/

U.S. Department of Agriculture. (n.d.). Healthy Eating for Women who are Pregnant or Breastfeeding. Accessed from: <https://www.myplate.gov/tip-sheet/healthy-eating-women-who-are-pregnant-or-breastfeeding>

PPD 101: Feeding Your Baby

| Cindy: I am having a difficult time feeding my baby. He is having trouble latching.  Elaine: The same thing happened to me! | 4.2 Information about antecedents |
| --- | --- |
| Elaine: I talked to my pediatrician and her lactation consultant. | 6.1 Demonstration of behavior |
| They gave me some excellent tips:   - Make myself comfortable when breastfeeding so that I could enjoy it more. - She taught me to recognize when Lucy was hungry. For example, if I saw that she was making sucking noises, I knew it was time for her to eat. - She told me to not use any bottles or pacifiers during the first few weeks of breastfeeding to avoid nipple confusion. | 4.1. Instruction on how to perform the behavior |
| Elaine: They also assured me that it would be ok if I decided to formula feed.  Cindy: I thought breastfeeding was better than formula!  Elaine: Breastfeeding does have some great benefits, but it is also important to consider how you feel about it. Formula can be a healthy alternative. | 5.1. Information about health  consequences |
| Cindy: Thank you for your advice! |  |

References:

Icahn School of Medicine at Mount Sinai. (2022). Breastfeeding vs. Formula Feeding. Accessed from: <https://www.mountsinai.org/health-library/special-topic/breastfeeding-vs-formula-feeding>

Nest Collaborative. (2021). Accessed from: <https://nestcollaborative.com>

Section on Breastfeeding (2012). Breastfeeding and the use of human milk. Pediatrics, 129(3), e827–e841. https://doi.org/10.1542/peds.2011-3552

Other evidence-based resources:

UT Physicians OB/GYN Offices (can request appointment with a lactation consultant)

https://www.utphysicians.com/specialty/obstetrics-and-gynecology/

832-325-7131

PPD 101: Family and Friends

| Jenny: My husband doesn’t understand the struggles that I’m going through as a new mom! I would just like for him to support me more with taking care of the baby. | 4.2 Information about antecedents |
| --- | --- |
| Therapist: New routines and responsibilities can be stressful. It is normal for couples to go through an adjustment phase as new parents. Keep in mind that fathers may also struggle. | 4.2 Information about antecedents  9.1. Credible source |
| Therapist: Some activities that might help you both reduce stress are:   - Schedule some time for just the two of you. It can be a simple activity like a 15-minute walk outside. - Doing fun activities as a family, like both of you playing with the baby. - Communicate how you are feeling, but without being aggressive. - Try to find ways to collaborate with taking care of the baby and around the house. For example, you can ask him what chores he would prefer to do. | 6.1 Demonstration of behavior  9.1. Credible source  3.2 Social Support (practical and emotional) |
| Jenny: Thank you! |  |

Evidenced-based resources:

Postpartum Support International. (2022). Help for partners and families. Accessed from: <https://www.postpartum.net/get-help/family/>

PPD 101: Everyday Tips for Mental Wellness

| Jenny: Hi Mary, can you tell me about things you do daily that make you feel good? |  |
| --- | --- |
| Mary: Hi Jenny! Yes, of course.  - I try to take at least 10-15 mins. of “me” time every day. I prefer mornings. Sometimes I write down, or just think about, three things that I am grateful for, or just enjoy the quiet.  - I listen to my favorite music while doing chores like cooking or cleaning.  - Connecting with other parents really helps! It makes me feel less lonely.  - Doing an outdoor activity is so refreshing to me! My doctor recommended that I go for 30-minute walks, at least 3-5 times a week. | 6.1 Demonstration of behavior |
| Jenny: This is very helpful! |  |
| Mary: And remember, it is important that you also keep up with your medications and doctor appointments. Any time you don’t feel well, don’t hesitate to speak up and ask for help if you need it! | BCT 3.2 Social Support (practical and emotional) |

References and Evidence-based resources:

Mothers and Babies. (n.d.). Self-Care Tips. Accessed from: <https://www.mothersandbabiesprogram.org/parents/self-care-tips/>

The American College of Obstetrics and Gynecology. (2020). Physical Activity and Exercise During Pregnancy and the Postpartum Period. Accessed from: https://www.acog.org/clinical/clinical-guidance/committee-opinion/articles/2020/04/physical-activity-and-exercise-during-pregnancy-and-the-postpartum-period
