# Supplementary material for: A pilot acceptability evaluation of MomMind: A digital health intervention for Peripartum Depression prevention and management focused on health disparities
Source: PLOS Digit Health. 2024 May 22;3(5):e0000508. doi: 10.1371/journal.pdig.0000508 (PMC11111021; doi:10.1371/journal.pdig.0000508)
Supplement: S2 Appendix — (DOCX) [file pdig.0000508.s002.docx]

**S2 Appendix**

Surveys and Questionnaires

Participant Survey

Q1 What is your age?

- 18-24 years old (1)
- 25-34 years old (2)
- 35-44 years old (3)
- 45+ years old (4)

Q3 What is your gender?

- Male (1)
- Female (2)
- Other (3) ________________________________________________
- Prefer not to answer (4)

Q4 Which best describes your race?

- White (1)
- Black or African American (2)
- American Indian or Alaska Native (3)
- Asian (4)
- Native Hawaiian or Pacific Islander (5)
- Other (6) ________________________________________________
- Prefer not to answer (7)

Q5 Are you Hispanic or Latino?

- Yes (1)
- No (2)

Q22 Education level (please select the highest you have completed)

- No schooling completed (1)
- Nursery school to 8th grade (2)
- Some high school, no diploma (3)
- High School Graduate, diploma or the equivalent (Example: GED) (4)
- Some college credit, no degree (5)
- Trade/Technical/Vocational training (6)
- Associate Degree (7)
- Bachelor's Degree (8)
- Master's Degree (9)
- Professional Degree (10)
- Doctoral Degree (11)

Q23 Employment Status

- Employed for wages (1)
- Self-employed (2)
- Out of work and looking for work (3)
- Out of work but not currently looking for work (4)
- Homemaker (5)
- Student (6)
- Military (7)
- Retired (8)
- Unable to work (9)
- Prefer not to answer (10)

Q6 What is your marital status?

- Single (Never Married) (1)
- Married (2)
- Widowed (3)
- Divorced (4)
- Separated (5)

Q24 Number of people currently living in your home:

________________________________________________________________

Q2 What is your annual household income?

- Under $20,000 (1)
- $20,001-$40,000 (2)
- $40,001-$60,000 (3)
- $60,001-$80,000 (4)
- Over $80,000 (5)

Q25 What languages do you speak in your home?

________________________________________________________________

Q26 What is your current Zip code?

________________________________________________________________

Q7 How many children do you have?

________________________________________________________________

Q8 How many pregnancies have you had?

________________________________________________________________

Q9 Are you currently pregnant?

- Yes (1)
- No (2)

Q10 How long ago did you have your most recent child?

________________________________________________________________

Q11 With your most recent/current pregnancy, have you experienced episodes of depression, feeling down, or what is sometimes called the baby blues?

- Yes (1)
- No (2)

*Display This Question:*

*If With your most recent/current pregnancy, have you experienced episodes of depression, feeling dow... = Yes*

Q13 When did you experience these episodes?

- During pregnancy (1)
- After delivery (2)
- Both (3)

*Display This Question:*

*If With your most recent/current pregnancy, have you experienced episodes of depression, feeling dow... = Yes*

Q16 Did you speak with a doctor about these feelings?

- Yes (1)
- No (2)

Q17 Did you have episodes of depression or feeling down with your previous pregnancies or following the birth of other children?

- Yes (1)
- No (2)

Q18 When you are interested in learning more about something like your pregnancy or how you are feeling do you:

- Look for information on the Internet (1)
- Speak with your Doctor (2)
- Look in books or other written source of information (3)
- Speak with friends or family (4)
- Look for information from social media (5)

Q19 Do you currently own a cell phone?

- Yes (1)
- No (2)

Q20 Do you use apps like:

- Games (1)
- Kindle (2)
- Spotify (3)
- Pregnancy related apps (4)
- Healthcare apps (tracking activity) (5)
- Banking apps (6)
- Uber/Lyft (7)
- Shopping apps (Amazon, Target, Walmart) (8)
- Netflix (9)
- Google Maps (10)
- Snapchat (11)
- Instagram (12)
- Facebook (13)
- Twitter (14)
- WhatsApp (15)

Q21 What is your favorite app?

Pick-a-Mood Survey


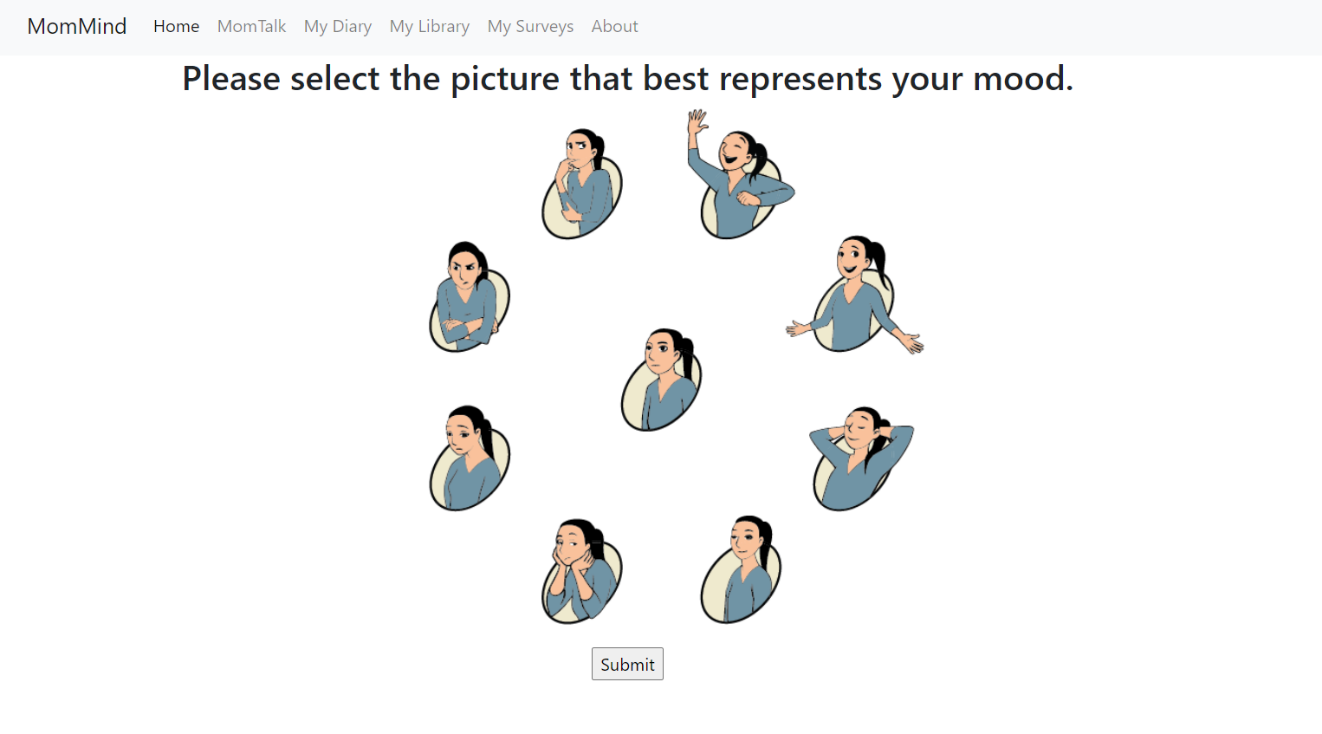


Edinburgh Postnatal Depression Scale

As you are pregnant or have recently had a baby, we would like to know how you are feeling. In the past 7 days:

I have been able to laugh and see the funny side of things

•As much as I always could

•Not quite so much now

•Definitely not so much now

•Not at all

I have looked forward with enjoyment to things

•As much as I ever did

•Rather less than I used to

•Definitely less than I used to

•Hardly at all

I have blamed myself unnecessarily when things went wrong

•Yes, most of the time

•Yes, some of the time

•Not very often

•No, never

I have been anxious or worried for no good reason

•No, not at all

•Hardly ever

•Yes, sometimes

•Yes, very often

I have felt scared or panicky for no very good reason

•Yes, quite a lot

•Yes, sometimes

•No, not much

•No, not at all

Things have been getting on top of me

•Yes, most of the time I haven't been able to cope at all

•Yes, sometimes I haven't been coping as well as usual

•No, most of the time I have coped quite well

•No, I have been coping as well as ever

I have been so unhappy that I have had difficulty sleeping

•Yes, most of the time

•Yes, sometimes

•Not very often

•No, not at all

I have felt sad or miserable

•Yes, most of the time

•Yes, quite often

•Not very often

•No, not at all

I have been so unhappy that I have been crying

•Yes, most of the time

•Yes, quite often

•Only ocassionally

•No, never

The thought of harming myself has occurred to me

•Yes, quite often

•Sometimes

•Hardly ever

•Never

Postpartum Depression Literacy Scale

Please rate your level of agreement with the following statements (1 = strongly disagree or not likely at all and 5 = strongly agree or very likely)

1. Feeling unusually sad and teary may be a symptom of postpartum depression
2. Sleeping too much or too little may be a sign of postpartum depression
3. Eating too much or losing interest in food may be a sign of postpartum depression
4. Loss of interest or pleasure in most things may be a symptom of postpartum depression
5. Postpartum depression affects person’s memory and concentration
6. Symptoms and signs of postpartum depression last for a period of at least 2 weeks
7. How likely is it that postpartum depression might be caused by a genetic or inherited problem
8. How likely is it that postpartum depression might be caused by stressful circumstances in the life (such as the death of a loved one or divorce)?
9. How likely is it that postpartum depression might be caused by lock of social support such as intimate partner support?
10. How likely is it that postpartum depression might be caused by a previous history of depression?
11. How likely is it that postpartum depression might be caused by a hormonal imbalance?
12. Physical activity is effective for the prevention or management of postpartum depression
13. Seeking help with tasks like infant care and house hold chores from intimate partners and family members is helpful for the prevention or management of postpartum depression
14. Religious practices, prayer and going to holy shrine are helpful for the prevention or management of postpartum depression
15. Having a balanced diet is helpful for the prevention or management of postpartum depression
16. Good sleep is helpful in prevention or management of postpartum depression
17. Although there are clinics for with postpartum depression, I would not have much faith in them
18. Treatment for postpartum depression, provided by a mental health professional, can be effective
19. Psychotherapy (for example, talking therapy or counselling) can be effective in treating postpartum depression
20. Antidepressants are addictive
21. Antidepressants cause brain damage
22. I would rather live with postpartum depression than go through the ordeal of getting psychiatric treatment
23. Most women who have postpartum depression are violent
24. It is best to avoid women with postpartum depression so that you don’t develop this problem
25. If I had postpartum depression I would not tell anyone
26. I am afraid of what my family and/or friends might think of me for attending psychology and/or psychiatry appointments
27. I know where to seek information about postpartum depression
28. I know how to use various sources to seek information about postpartum depression
29. I can appraise the accuracy of information about postpartum depression on the radio and television
30. I can appraise the accuracy of information about postpartum depression on the Internet
31. I can appraise the accuracy of advices about postpartum depression which given me by friends and family members
