# Supplementary material for: A pilot acceptability evaluation of MomMind: A digital health intervention for Peripartum Depression prevention and management focused on health disparities
Source: PLOS Digit Health. 2024 May 22;3(5):e0000508. doi: 10.1371/journal.pdig.0000508 (PMC11111021; doi:10.1371/journal.pdig.0000508)
Supplement: S3 Appendix — (PPTX) [file pdig.0000508.s003.pptx]

## Slide 1
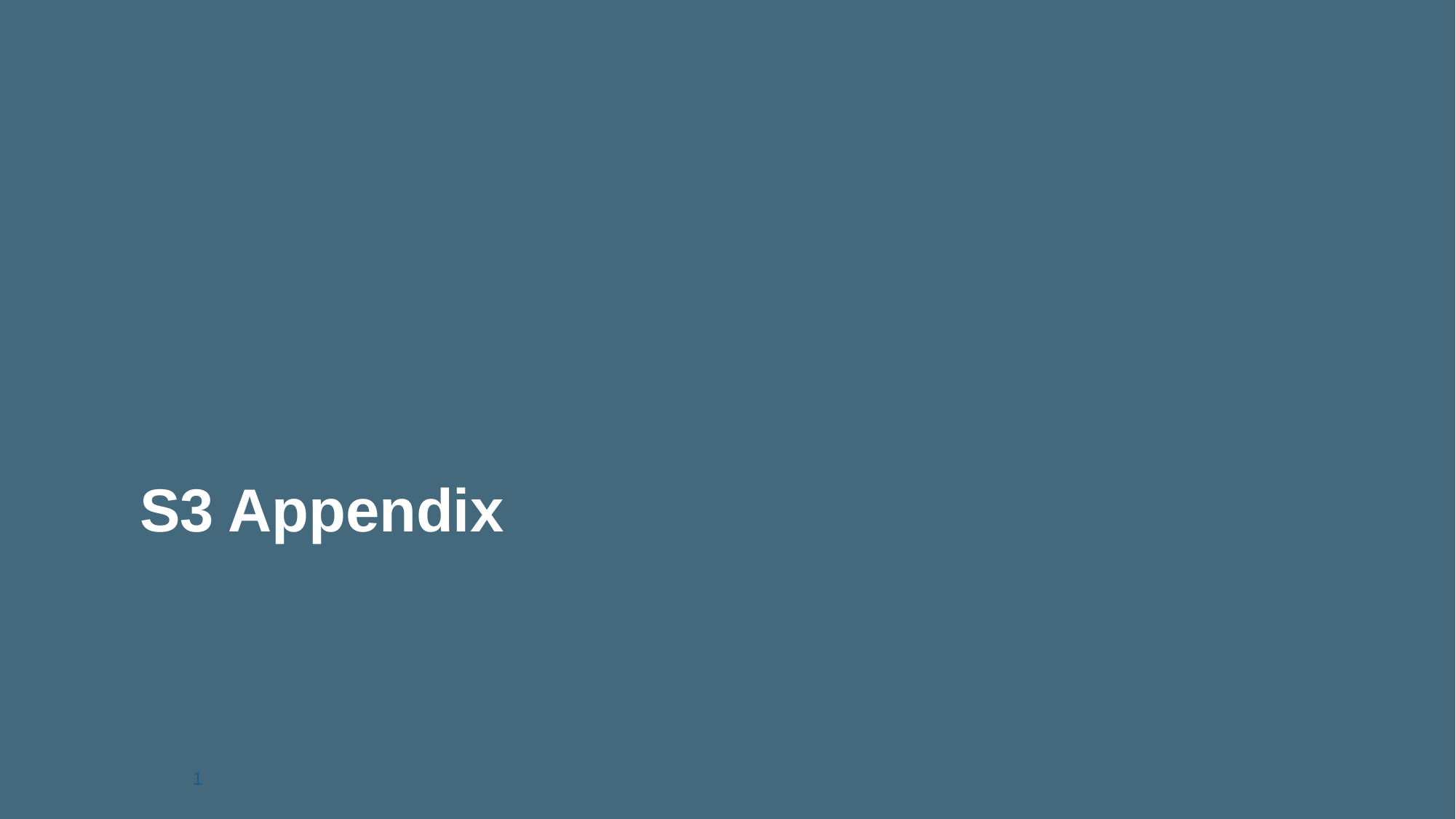

# S3 Appendix
1

## Slide 2
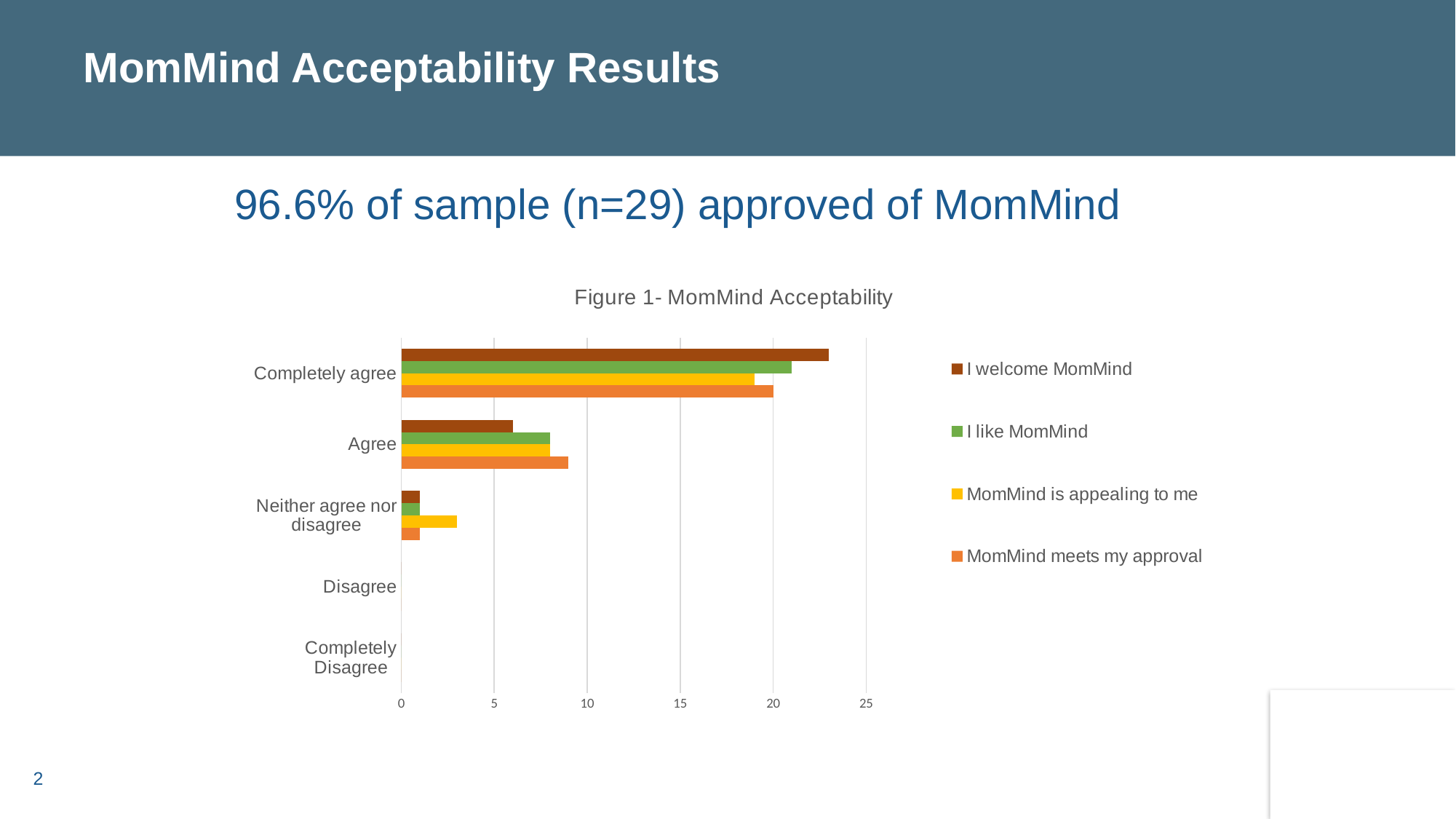

# MomMind Acceptability Results
96.6% of sample (n=29) approved of MomMind
### Chart: Figure 1- MomMind Acceptability
| Category | MomMind meets my approval | MomMind is appealing to me | I like MomMind | I welcome MomMind |
|---|---|---|---|---|
| Completely Disagree | 0.0 | 0.0 | 0.0 | 0.0 |
| Disagree | 0.0 | 0.0 | 0.0 | 0.0 |
| Neither agree nor disagree | 1.0 | 3.0 | 1.0 | 1.0 |
| Agree | 9.0 | 8.0 | 8.0 | 6.0 |
| Completely agree | 20.0 | 19.0 | 21.0 | 23.0 |
2

## Slide 3
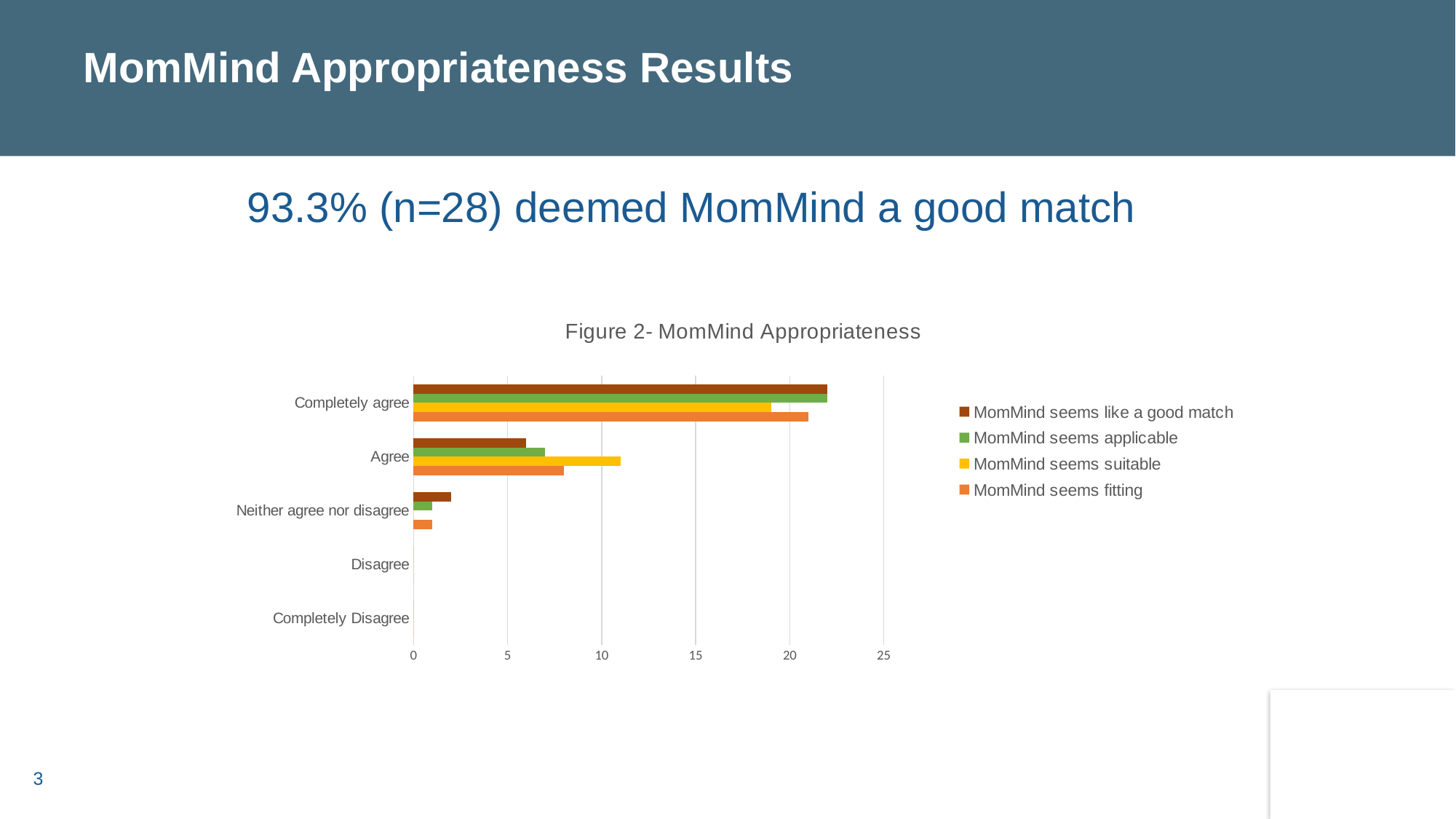

# MomMind Appropriateness Results
93.3% (n=28) deemed MomMind a good match
### Chart: Figure 2- MomMind Appropriateness
| Category | MomMind seems fitting | MomMind seems suitable | MomMind seems applicable | MomMind seems like a good match |
|---|---|---|---|---|
| Completely Disagree | 0.0 | 0.0 | 0.0 | 0.0 |
| Disagree | 0.0 | 0.0 | 0.0 | 0.0 |
| Neither agree nor disagree | 1.0 | 0.0 | 1.0 | 2.0 |
| Agree | 8.0 | 11.0 | 7.0 | 6.0 |
| Completely agree | 21.0 | 19.0 | 22.0 | 22.0 |
3

## Slide 4
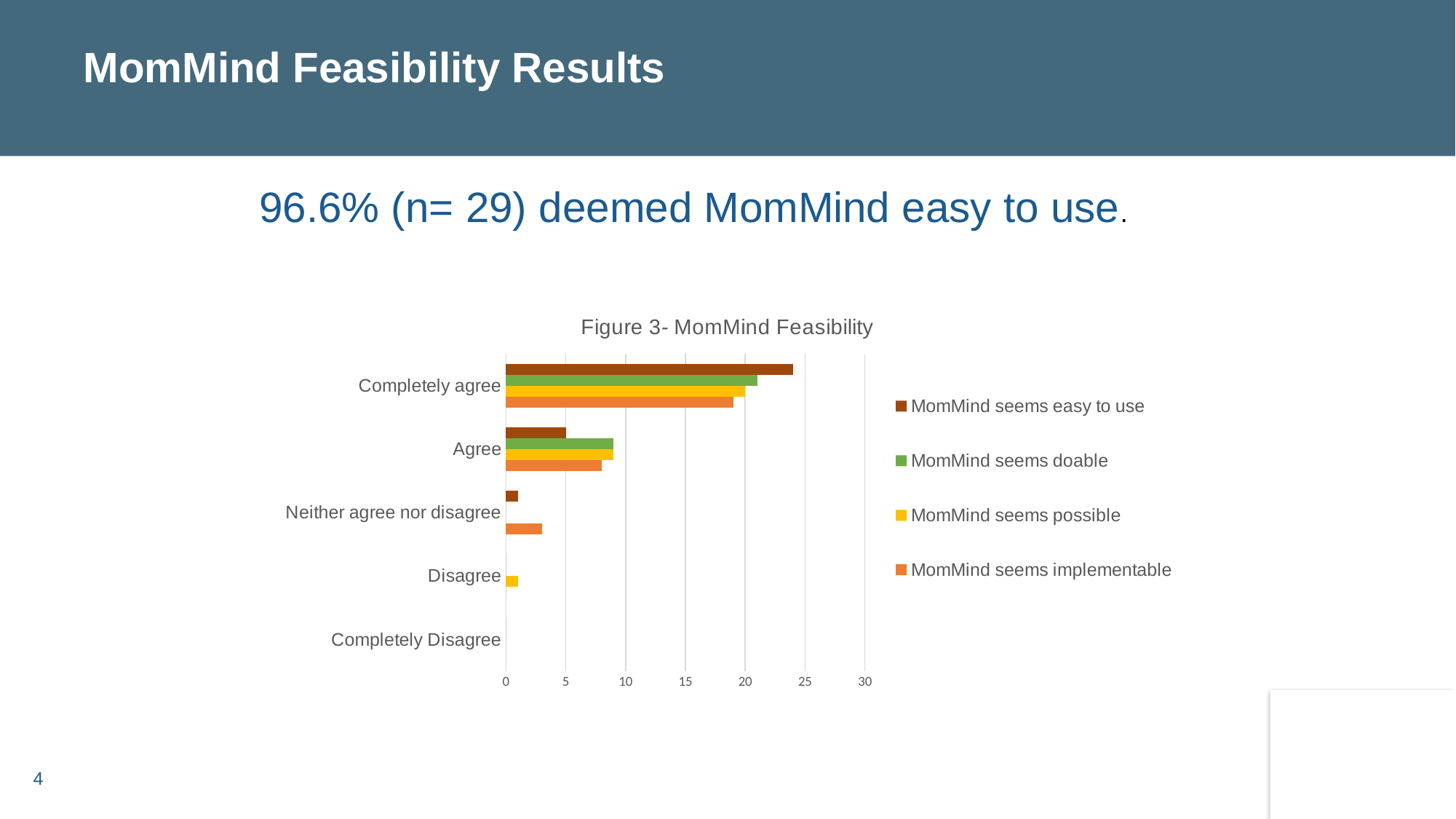

# MomMind Feasibility Results
96.6% (n= 29) deemed MomMind easy to use.
### Chart: Figure 3- MomMind Feasibility
| Category | MomMind seems implementable | MomMind seems possible | MomMind seems doable | MomMind seems easy to use |
|---|---|---|---|---|
| Completely Disagree | 0.0 | 0.0 | 0.0 | 0.0 |
| Disagree | 0.0 | 1.0 | 0.0 | 0.0 |
| Neither agree nor disagree | 3.0 | 0.0 | 0.0 | 1.0 |
| Agree | 8.0 | 9.0 | 9.0 | 5.0 |
| Completely agree | 19.0 | 20.0 | 21.0 | 24.0 |
4

## Slide 5
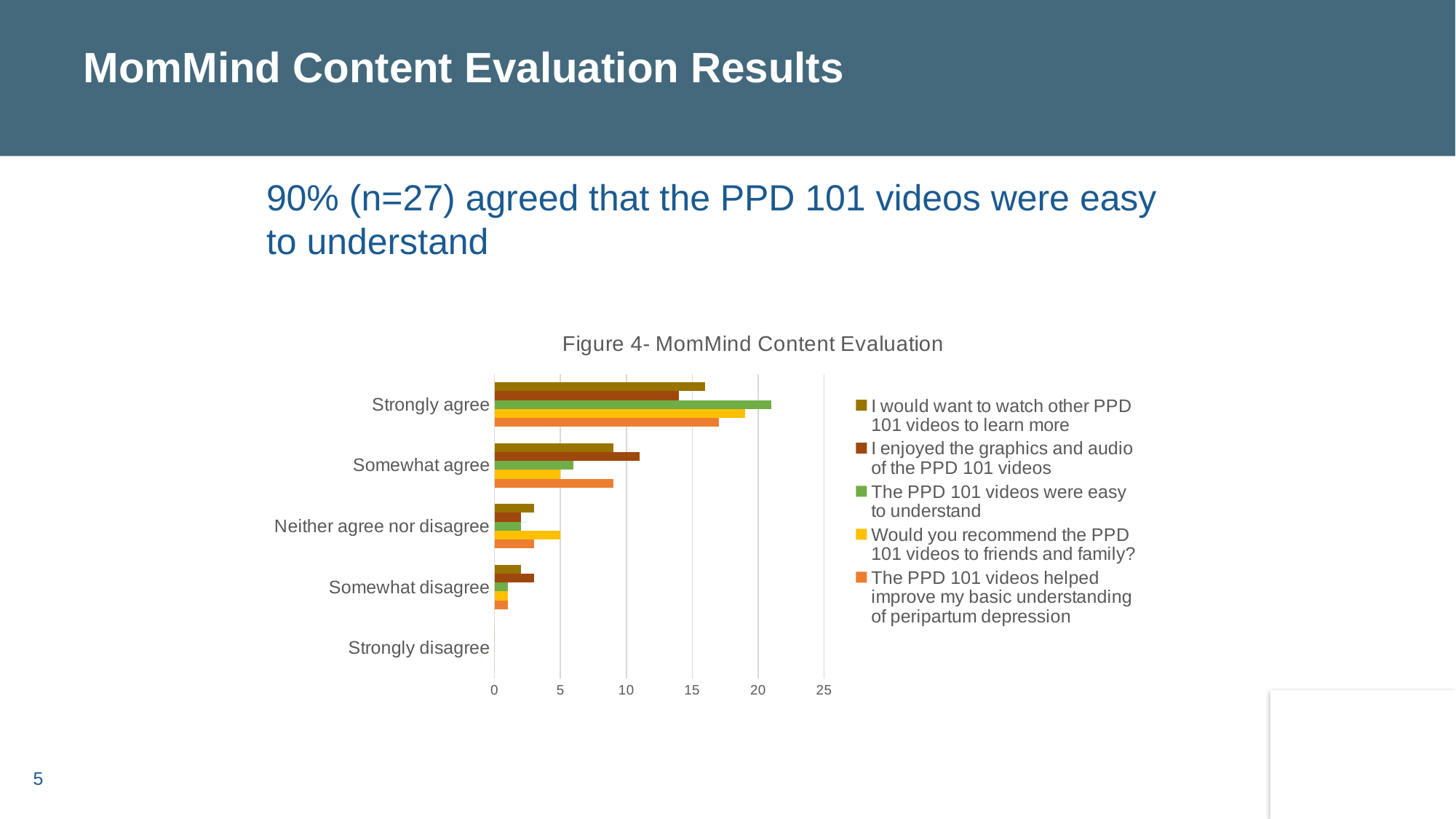

# MomMind Content Evaluation Results
90% (n=27) agreed that the PPD 101 videos were easy to understand
### Chart: Figure 4- MomMind Content Evaluation
| Category | The PPD 101 videos helped improve my basic understanding of peripartum depression | Would you recommend the PPD 101 videos to friends and family? | The PPD 101 videos were easy to understand | I enjoyed the graphics and audio of the PPD 101 videos | I would want to watch other PPD 101 videos to learn more |
|---|---|---|---|---|---|
| Strongly disagree | 0.0 | 0.0 | 0.0 | 0.0 | 0.0 |
| Somewhat disagree | 1.0 | 1.0 | 1.0 | 3.0 | 2.0 |
| Neither agree nor disagree | 3.0 | 5.0 | 2.0 | 2.0 | 3.0 |
| Somewhat agree | 9.0 | 5.0 | 6.0 | 11.0 | 9.0 |
| Strongly agree | 17.0 | 19.0 | 21.0 | 14.0 | 16.0 |
5

## Slide 6
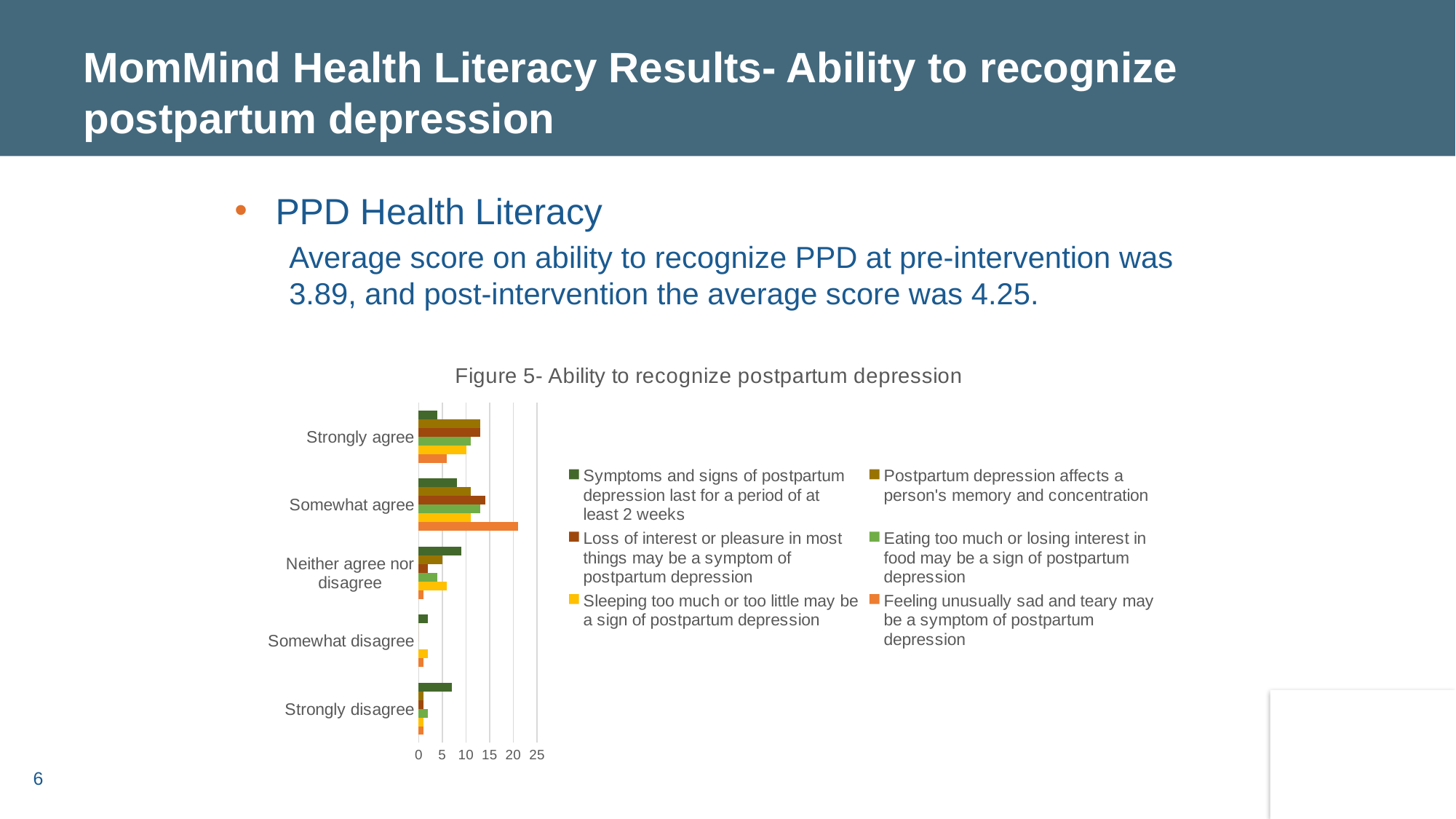

# MomMind Health Literacy Results- Ability to recognize postpartum depression
PPD Health Literacy
Average score on ability to recognize PPD at pre-intervention was 3.89, and post-intervention the average score was 4.25.
### Chart: Figure 5- Ability to recognize postpartum depression
| Category | Feeling unusually sad and teary may be a symptom of postpartum depression | Sleeping too much or too little may be a sign of postpartum depression | Eating too much or losing interest in food may be a sign of postpartum depression | Loss of interest or pleasure in most things may be a symptom of postpartum depression | Postpartum depression affects a person's memory and concentration | Symptoms and signs of postpartum depression last for a period of at least 2 weeks |
|---|---|---|---|---|---|---|
| Strongly disagree | 1.0 | 1.0 | 2.0 | 1.0 | 1.0 | 7.0 |
| Somewhat disagree | 1.0 | 2.0 | 0.0 | 0.0 | 0.0 | 2.0 |
| Neither agree nor disagree | 1.0 | 6.0 | 4.0 | 2.0 | 5.0 | 9.0 |
| Somewhat agree | 21.0 | 11.0 | 13.0 | 14.0 | 11.0 | 8.0 |
| Strongly agree | 6.0 | 10.0 | 11.0 | 13.0 | 13.0 | 4.0 |
6

## Slide 7
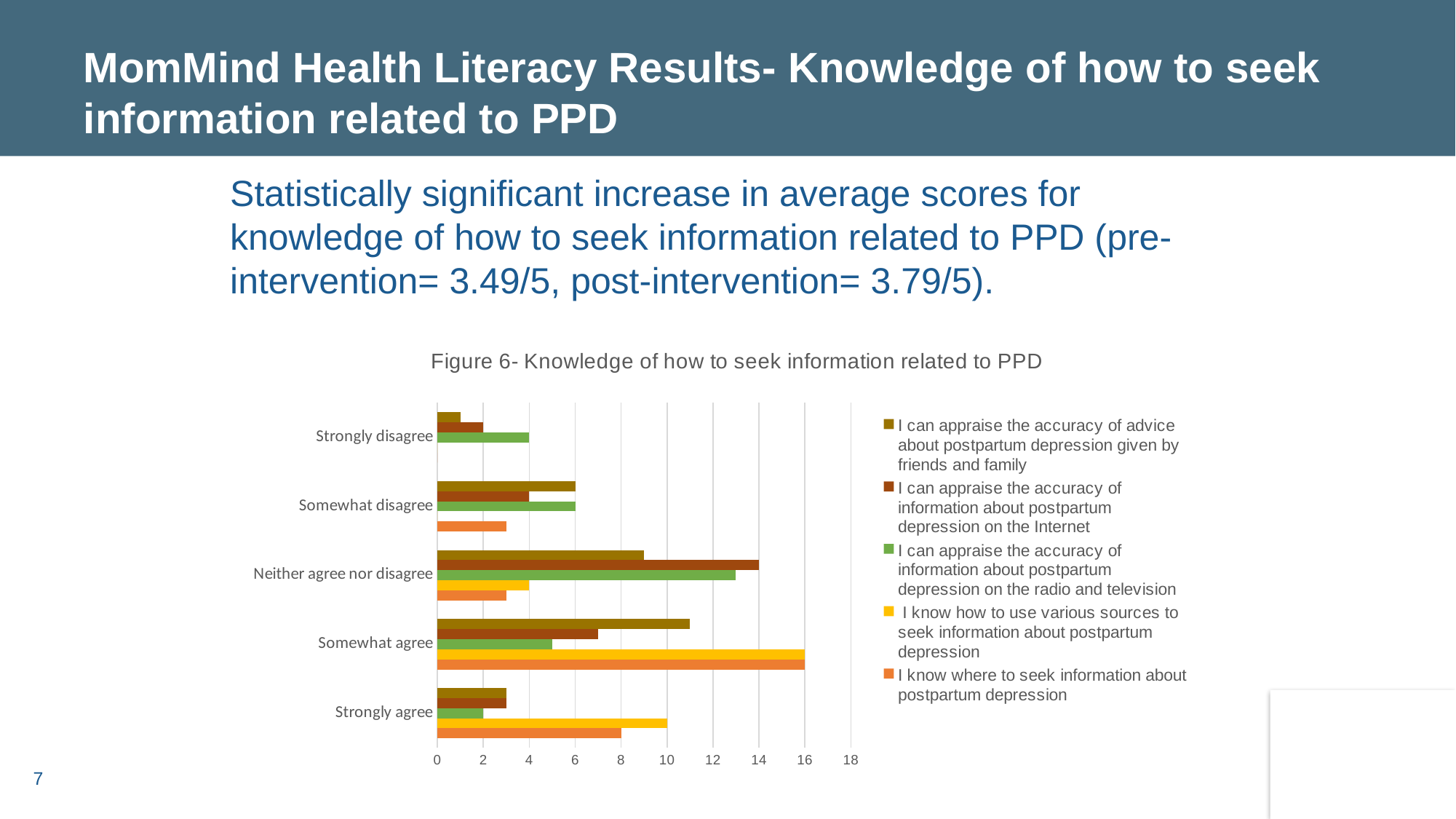

# MomMind Health Literacy Results- Knowledge of how to seek information related to PPD
Statistically significant increase in average scores for knowledge of how to seek information related to PPD (pre-intervention= 3.49/5, post-intervention= 3.79/5).
### Chart: Figure 6- Knowledge of how to seek information related to PPD
| Category | I know where to seek information about postpartum depression | I know how to use various sources to seek information about postpartum depression | I can appraise the accuracy of information about postpartum depression on the radio and television | I can appraise the accuracy of information about postpartum depression on the Internet | I can appraise the accuracy of advice about postpartum depression given by friends and family |
|---|---|---|---|---|---|
| Strongly agree | 8.0 | 10.0 | 2.0 | 3.0 | 3.0 |
| Somewhat agree | 16.0 | 16.0 | 5.0 | 7.0 | 11.0 |
| Neither agree nor disagree | 3.0 | 4.0 | 13.0 | 14.0 | 9.0 |
| Somewhat disagree | 3.0 | 0.0 | 6.0 | 4.0 | 6.0 |
| Strongly disagree | 0.0 | 0.0 | 4.0 | 2.0 | 1.0 |
7

## Slide 8
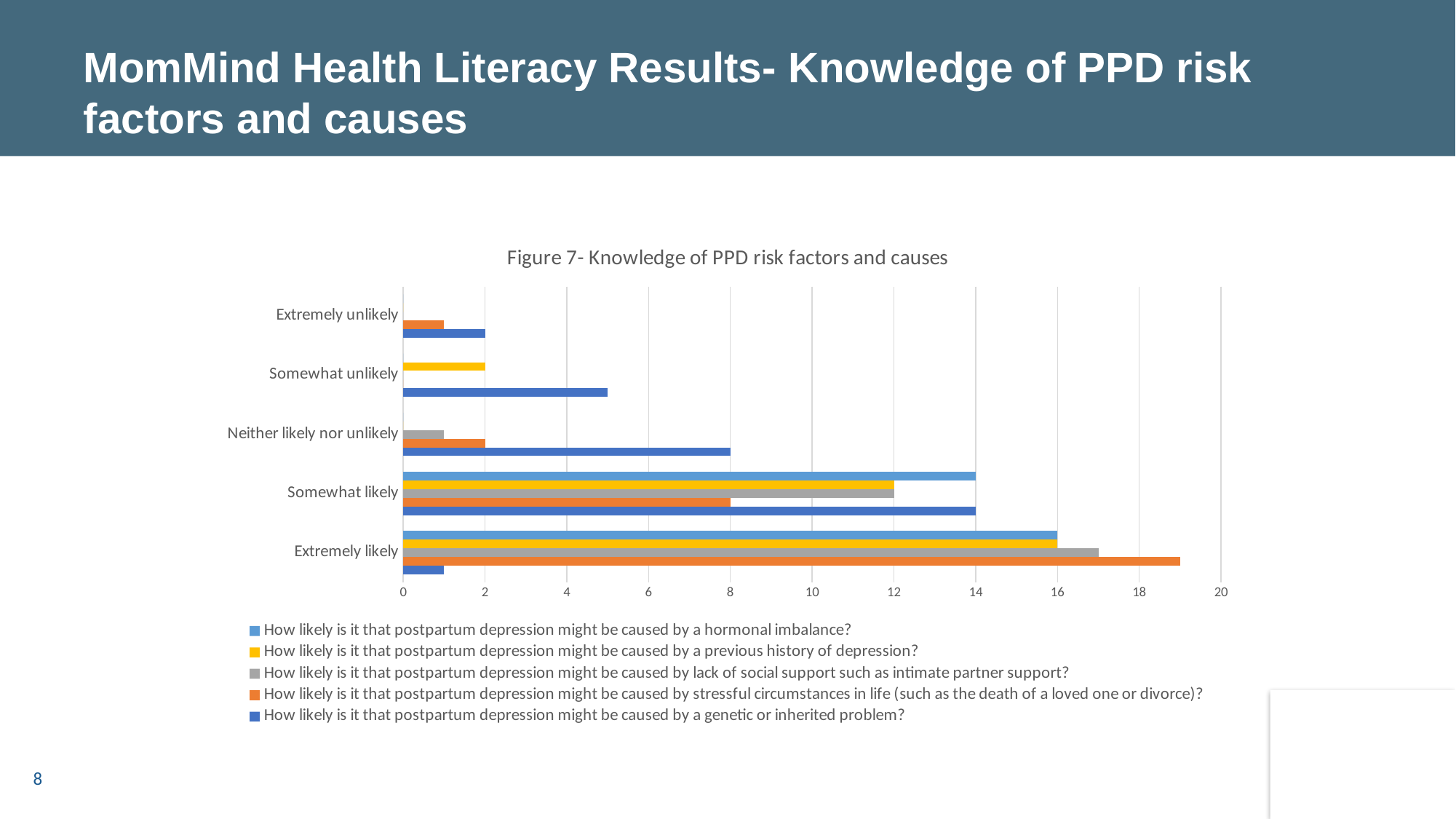

# MomMind Health Literacy Results- Knowledge of PPD risk factors and causes
### Chart: Figure 7- Knowledge of PPD risk factors and causes
| Category | How likely is it that postpartum depression might be caused by a genetic or inherited problem? | How likely is it that postpartum depression might be caused by stressful circumstances in life (such as the death of a loved one or divorce)? | How likely is it that postpartum depression might be caused by lack of social support such as intimate partner support? | How likely is it that postpartum depression might be caused by a previous history of depression? | How likely is it that postpartum depression might be caused by a hormonal imbalance? |
|---|---|---|---|---|---|
| Extremely likely | 1.0 | 19.0 | 17.0 | 16.0 | 16.0 |
| Somewhat likely | 14.0 | 8.0 | 12.0 | 12.0 | 14.0 |
| Neither likely nor unlikely | 8.0 | 2.0 | 1.0 | 0.0 | 0.0 |
| Somewhat unlikely | 5.0 | 0.0 | 0.0 | 2.0 | 0.0 |
| Extremely unlikely | 2.0 | 1.0 | 0.0 | 0.0 | 0.0 |
8

## Slide 9
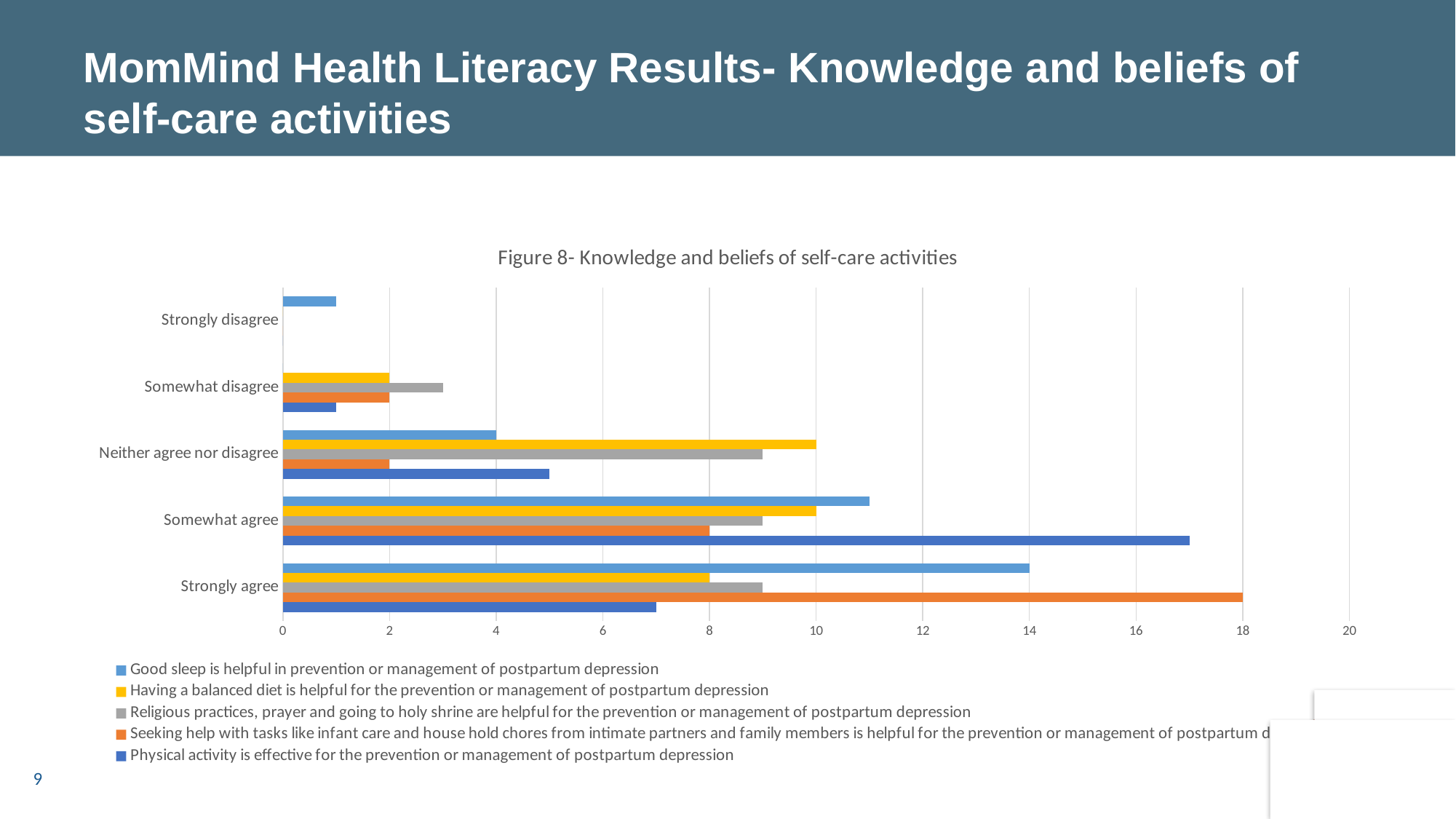

# MomMind Health Literacy Results- Knowledge and beliefs of self-care activities
### Chart: Figure 8- Knowledge and beliefs of self-care activities
| Category | Physical activity is effective for the prevention or management of postpartum depression | Seeking help with tasks like infant care and house hold chores from intimate partners and family members is helpful for the prevention or management of postpartum depression | Religious practices, prayer and going to holy shrine are helpful for the prevention or management of postpartum depression | Having a balanced diet is helpful for the prevention or management of postpartum depression | Good sleep is helpful in prevention or management of postpartum depression |
|---|---|---|---|---|---|
| Strongly agree | 7.0 | 18.0 | 9.0 | 8.0 | 14.0 |
| Somewhat agree | 17.0 | 8.0 | 9.0 | 10.0 | 11.0 |
| Neither agree nor disagree | 5.0 | 2.0 | 9.0 | 10.0 | 4.0 |
| Somewhat disagree | 1.0 | 2.0 | 3.0 | 2.0 | 0.0 |
| Strongly disagree | 0.0 | 0.0 | 0.0 | 0.0 | 1.0 |
9

## Slide 10
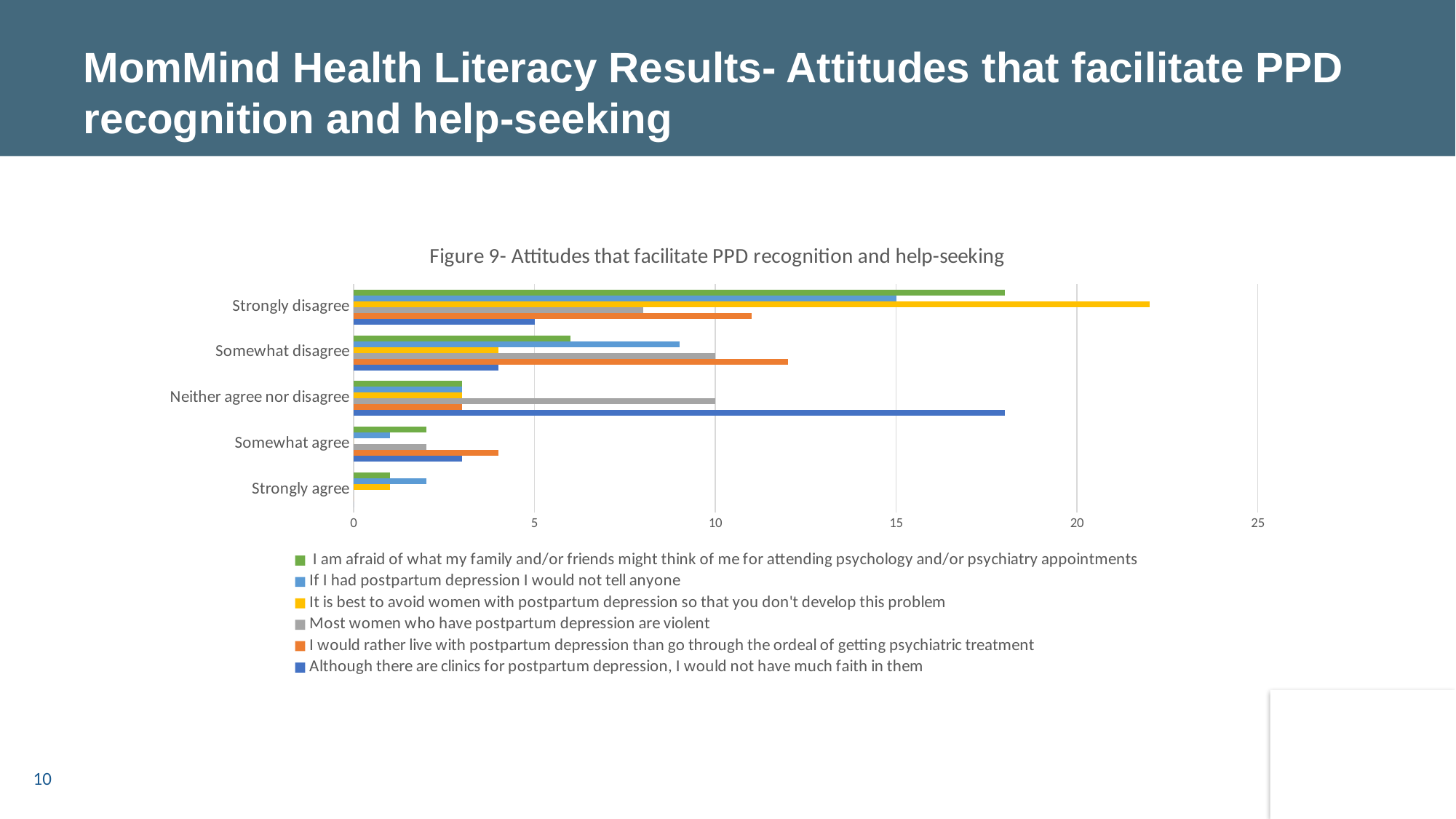

# MomMind Health Literacy Results- Attitudes that facilitate PPD recognition and help-seeking
### Chart: Figure 9- Attitudes that facilitate PPD recognition and help-seeking
| Category | Although there are clinics for postpartum depression, I would not have much faith in them | I would rather live with postpartum depression than go through the ordeal of getting psychiatric treatment | Most women who have postpartum depression are violent | It is best to avoid women with postpartum depression so that you don't develop this problem | If I had postpartum depression I would not tell anyone | I am afraid of what my family and/or friends might think of me for attending psychology and/or psychiatry appointments |
|---|---|---|---|---|---|---|
| Strongly agree | 0.0 | 0.0 | 0.0 | 1.0 | 2.0 | 1.0 |
| Somewhat agree | 3.0 | 4.0 | 2.0 | 0.0 | 1.0 | 2.0 |
| Neither agree nor disagree | 18.0 | 3.0 | 10.0 | 3.0 | 3.0 | 3.0 |
| Somewhat disagree | 4.0 | 12.0 | 10.0 | 4.0 | 9.0 | 6.0 |
| Strongly disagree | 5.0 | 11.0 | 8.0 | 22.0 | 15.0 | 18.0 |
10

## Slide 11
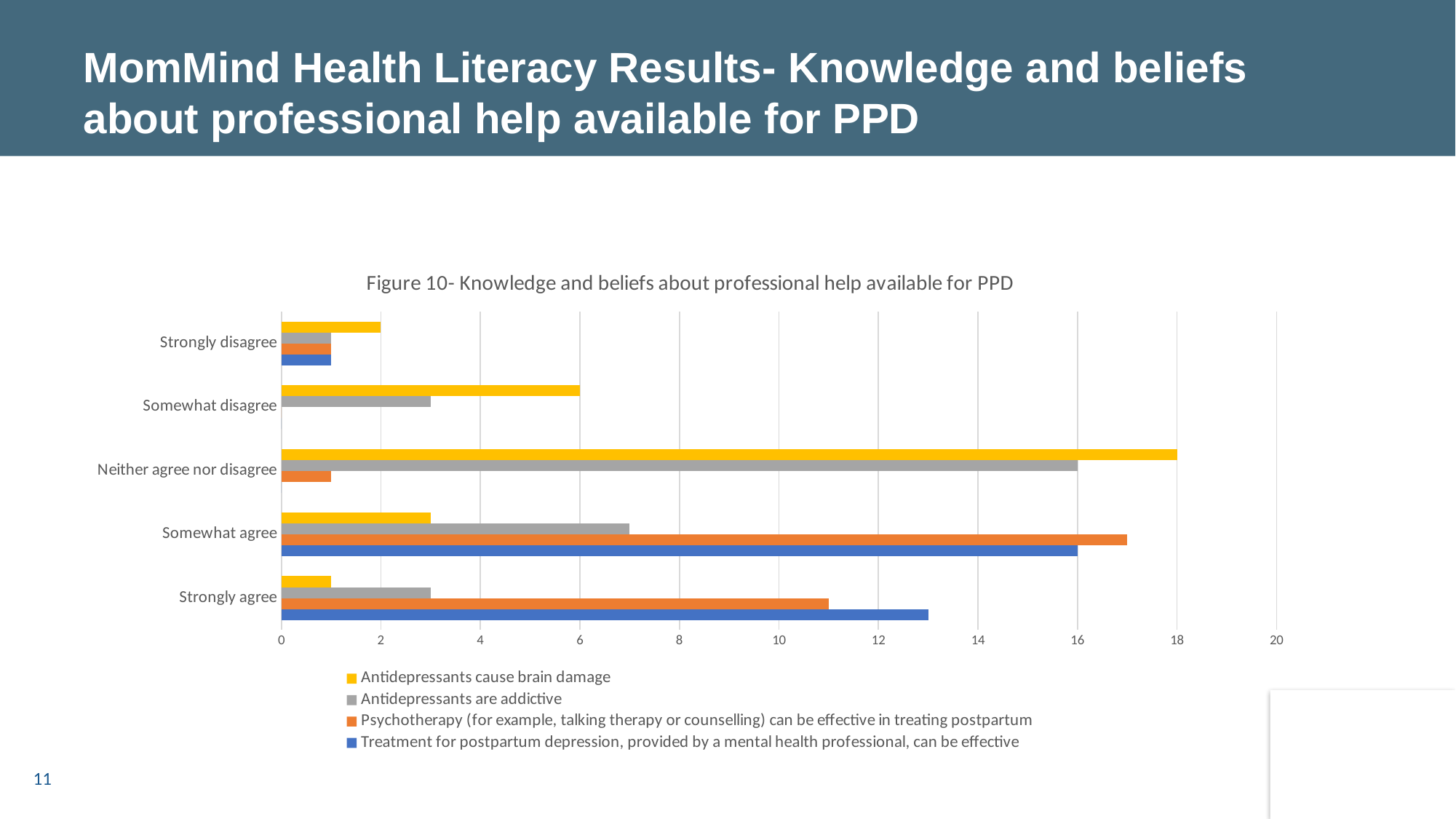

# MomMind Health Literacy Results- Knowledge and beliefs about professional help available for PPD
### Chart: Figure 10- Knowledge and beliefs about professional help available for PPD
| Category | Treatment for postpartum depression, provided by a mental health professional, can be effective | Psychotherapy (for example, talking therapy or counselling) can be effective in treating postpartum | Antidepressants are addictive | Antidepressants cause brain damage |
|---|---|---|---|---|
| Strongly agree | 13.0 | 11.0 | 3.0 | 1.0 |
| Somewhat agree | 16.0 | 17.0 | 7.0 | 3.0 |
| Neither agree nor disagree | 0.0 | 1.0 | 16.0 | 18.0 |
| Somewhat disagree | 0.0 | 0.0 | 3.0 | 6.0 |
| Strongly disagree | 1.0 | 1.0 | 1.0 | 2.0 |
11
