# Supplementary material for: A pilot acceptability evaluation of MomMind: A digital health intervention for Peripartum Depression prevention and management focused on health disparities
Source: PLOS Digit Health. 2024 May 22;3(5):e0000508. doi: 10.1371/journal.pdig.0000508 (PMC11111021; doi:10.1371/journal.pdig.0000508)
Supplement: Attachment — Submitted filename: Response to Reviewers.docx [file pdig.0000508.s005.docx]

We would like to thank all reviewers for their constructive feedback. We are now submitting a revised version of our manuscript based on this feedback. Please see our point-by-point response to reviewer comments:

1) Reviewer #1: Thank you for the opportunity to review this paper. This is an interesting study and a nice pilot outcomes paper. My suggestions are as follows;

Please consider shortening the intro from pages 3-6, where it is essentially describing the previously published paper. The descriptions about the software and other detailed information is not needed here and is replicating previous work. The key elements of the intervention can be presented in a table, briefly summarised referencing previous papers, and then clearly stating what the gap this paper is filling. The last sentences of the introduction need to include the gap in the literature and the aim of this study and expected outcomes.

*We have shortened the Introduction as suggested by removing details on the software and other previous work (please see Pages 2-3). We have also created a table summarizing key elements of the intervention (Table 1, Pg. 3). And, please see our new sentences at the end of the introduction stating the gap in literature, aim of the study, and expected outcomes (Pg. 3, Par. 1).*

methods: please justify the sample size, for the quantitative outcomes and the qualitative outcomes.

*We have added justification of our sample size as follows: “Our study sample size is in accordance with statistical recommendations indicating that sample sizes between approximately n=26 to n=34 are satisfactory for studies gauging initial acceptability/feasibility of a process or product, as the emphasis of such studies is to capture initial trends, preliminary insights, and qualitative feedback rather than statistical significance (16)” (Pg. 4, Par. 1)*

*New Reference:*

*16. Lewis M, Bromley K, Sutton CJ, McCray G, Myers HL, Lancaster GA. Determining sample size for progression criteria for pragmatic pilot RCTs: the hypothesis test strikes back! Pilot and Feasibility Studies. 2021;7(1):40.*

The section under the heading Evaluation Procedures can be collapsed into a single table

*Please see our new Table 2 summarizing the evaluation procedures (Pg. 5)*

Please present a figure showing the steps of the study, the study procedure, and then revisit the wording to see what can be stripped back as the method is very long.

*We have introduced a new figure representing our methodology (Figure 1) and revised the wording in our methods as suggested (Pg. 3, Par. 2).*

Demographic data can be presented in a table (page 12), again improving readability.

*Please see our new Table 3 presenting participants’ demographic data (Pg. 8)*

The whole section on the interview themes is also well suited to a table, improving readability. (pages 14-16).

*Please see Table 4 (Pg. 11)*

Please start the discussion with the aim of the study

*We are now starting our discussion by stating the aim of the study: “This study aims to assess the level of acceptability a PPD digital health tool developed through a framework focused on health disparities will have among low-income perinatal women” (Pg. 13, Par. 2).*

The point that: Future digital health interventions targeting vulnerable populations should consider the use of specialized digital health frameworks addressing social determinants of health such as health literacy (an example being the extended Digilego framework we have presented in this study) for intervention design and development - is excellent. Can you refer to literature where this has been done? Can you expand on the consequences of not doing this?

*Thank you for this very useful suggestion. We have now extended our conclusion paragraph to address these points:*

*“**Previous studies that also presented digital health tools targeting vulnerable populations have used approaches such as participatory design (29) and community-based participatory research (3). Other studies have also employed the eHealth literacy framework during development of digital interventions in areas ranging from nutrition and aging (30) to non-communicable diseases (31). While these represent the correct direction towards digital health equity, there is still a significant scarcity of research that considers the underlying theories behind the development of these particular tools, a factor that can diminish the effectiveness of these tools and which can contribute to an existing digital health divide (32)” (Page 15, par. 1).*

*New References:*

*30.	Nakamura S, Inayama T, Arao T. A randomized-controlled trial focusing on socio-economic status for promoting vegetable intake among adults using a web-based nutrition intervention programme: study protocol. BMC Public Health. 2017;17(1):74.*

*31.	Blalock SJ SE, Nowell WB, Woloshin S, Schwartz L, Carpenter DM, Curtis JR, Moreland LW, Hunt C, Hickey G, Reyna V. A Trial Testing Strategies to Enhance Patient Understanding of Drug Information: Experience Recruiting Subjects through an Online Patient Community [abstract]. Arthritis and Rheumatology,.69.*

*32.	El Benny M, Kabakian-Khasholian T, El-Jardali F, Bardus M. Application of the eHealth Literacy Model in Digital Health Interventions: Scoping Review. J Med Internet Res. 2021;23(6):e23473.*

2) Reviewer #2: Manuscript comments

- Including a table of participant characteristics would be very helpful, rather than reporting everything in the text. It would make the information easier to process.

*Please see Table 3, Pg. 8*

- How do you know your sample is representative of low-income women? Your target population is low-income perinatal women but your sample includes women who are not low-income.

*We have now clarified that our sample consists of women who in their majority are from low-income households (Pg. 3, par. 1).*

- If the purpose of MomMind is to eventually be launched and used by the target population, will the digital features of MomMind only be website-based or will they also be available on a smartphone as an app? It would be good to describe the future plans for MomMind, especially since the target population may not have easy access to a computer or smartphone. If the purpose of the study was to only show the acceptability/utility of such a tool then this should be stated.

*Thank you for this suggestion. We have now extended our discussion by describing our future plans for MomMind (please see Pg. 14, par. 3), which include recruitment of Spanish-speaking participants, adapting content for various cultures and languages, pursuing Randomized Controlled Trial studies, and eventual implementation.*

- Were all distributions skewed? It might be nice to know this as justification for using the Wilcoxon test.

*Yes, due to the Postpartum Depression Literacy Scale being based on Likert scale responses, the distribution for this instrument is skewed and justifies our use of the Wilcoxon signed-rank test for analysis of pre-post results. We now state this in Pg. 7, par. 4.*

*New Reference for justification:*

*23. Roberson PK, Shema SJ, Mundfrom DJ, Holmes TM. Analysis of paired Likert data: how to evaluate change and preference questions. Fam Med. 1995;27(10):671-5.*

Text editing comments

*Thank you for the text editing comments. We have followed all suggestions excepting the first two, for which the sections were revised or removed based on comments from other reviewers.*

- Need to cite Digilego in first sentence of “MomMind: A Health-Disparities Focused Digital Health Solution for PPD Self-Management” section

- In first sentence in Methods section, remove the word “has” from this line: Our mixed-methods study has involved the following steps:

- Typo in last sentence of Individual Interviews section: “intervies” should be “interviews”

- Same section, b) Instrumental Attitude: “and” should be “an”

- Same section, e) remove period at the end (after the question mark)

- In Peripartum Depression Health Literacy section, sentence “Average score on knowledge of PPD risk factors and causes at pre-intervention was 4.23, and at post-intervention 4.27, a change that is statistically unsignificant.” “unsignificant” should be “insignificant”

3) Reviewer #3: My Understanding of the Manuscript’s Intent:

The goal of the study was to evaluate the acceptability of a digital health tool called MomMind among low-income and minority women for managing peripartum depression. This pilot study aims to demonstrate the promising acceptability of a tailored digital health intervention for peripartum depression in underserved populations. The authors suggest enhancing design approaches for health equity.

My Review:

Given the interesting topic, solid evidence, and clear writing style, I would recommend the authors proceed with a submission to PLOS Digital Health after revising the data availability information and statistical analysis sections.

*Thank you for the kind words.*

Originality - The study presents novel research evaluating a tailored digital health tool for an underserved population. The integration of behavior change and health literacy frameworks is an original approach.

Importance & Interest - Peripartum depression interventions for vulnerable groups are an important topic with broad appeal to digital health researchers and clinicians.

Rigor - The mixed methods evaluation uses appropriate surveys, interviews, and analyses. The sample size is decent for a pilot study but ultimately is small for power/generalizability.

Evidence - Results provide evidence that the tailored MomMind tool had high acceptability and improved women's depression knowledge. Qualitative data further supports the tool's benefits.

Utility & Accessibility - The tool aims to promote accessibility of peripartum depression resources for underserved women. The open-access publication benefits the broader community.

Open Science - The study methods and results are reported in enough detail to be reproduced. Data availability statements indicate data can be accessed upon request. The study involves human subjects research, which can justify data access restrictions to protect privacy. Overall, the authors have not yet fully met the data availability requirements due to lack of details and limiting access to just the corresponding author.

A Note on Statistical Analysis

The analysis in this study appears to have been carried out appropriately and with sufficient rigor. The analytic methods align with the study design and data characteristics. The analyses appear to have been conducted appropriately without obvious deficiencies. The statistical rigor is sufficient for the aims and scale of this pilot study. The reporting of methods and results is reasonably comprehensive and transparent. The analysis methods align with the study aims and data types. Using descriptive statistics, Wilcoxon signed-rank tests, and qualitative coding is appropriate, especially for a sample size of 30 participants since normal distribution cannot be assumed. The results section provides adequate numeric details from the analyses. Some key limitations around sample size and follow-up time are acknowledged. Changes in depression knowledge could be influenced by factors outside MomMind like prenatal classes. Controlling for confounders would strengthen conclusions.

There is no control or comparison group, limiting the ability to attribute results solely to MomMind. The pre-post, non-randomized design cannot determine causality or directionality. Greater rigor would be experimental or RCT designs.

*Thank you for the suggestion. We have now included this in our future plans for MomMind in the Discussion section (Page 14, Par 3).*

A Note on Data Access:

To properly comply, the authors should edit their data availability statement to:

-	Provide an institutional/committee contact for data requests.

-	Specify in detail the reasons and ethical approvals for restricted access.

-	Clarify what specific data cannot be public and what could potentially be shared in an anonymous form.

*After consulting with the UTHealth Committee for the Protection of Human Subjects, we have been granted permission to share de-identified study data. Therefore, we have now revised our Data Access statement as follows:* “*The data to reproduce the results presented are included in Supporting Information files.”*

An Additional Note on Equity:

The study focuses on evaluating a digital health tool specifically designed for an underserved population - low-income and minority women at risk for peripartum depression. This aligns well with the goals of promoting equity. The introduction highlights the disproportionate burden of peripartum depression in minority and low-SES groups as a motivation for the research.

-	Within the remit of the US-based work, intersectionality could be better addressed - such as mental health access issues for low-income Hispanic women specifically, and an additional lens on potential barriers like digital literacy, disability, etc. that could affect. Incorporate perspectives from women facing multiple disparities. Discuss how UI design, literacy level, etc. were optimized for inclusivity.

*Thank you for the suggestion. We have now described the various barriers that low-income Hispanic women may experience in our Discussion section, such as language barriers (Pg. 14, par. 3). We also discuss how we optimized MomMind for inclusivity through participation of vulnerable women in our user needs analysis and developing “PPD 101” content that is easy to process and representative of our target population (Pg. 13, par. 2).*

*New References for this section of the discussion:*

*27.	Barrera AZ, Nichols AD. Depression help-seeking attitudes and behaviors among an Internet-based sample of Spanish-speaking perinatal women. Revista panamericana de salud publica. 2015;37:148-53.*

*28.	Fryer K, Lewis G, Munoz C, Stuebe AM. Identifying Barriers and Facilitators to Prenatal Care for Spanish-Speaking Women. N C Med J. 2021;82(1):7-13.*

-	Nice to have for the future, the study sample is US-based, limiting (global) generalizability. Discuss how the digital health framework could be adapted for diverse cultural contexts. While not entirely within the remit of the pilot study, consider partnerships for evaluating the tool in low-resource global health settings. Low-income regions may have lower access to mobile devices needed to use the tool.

*We agree with these suggestions and we now describe how future plans for MomMind include adapting it for various cultures and the possibility of partnering with research colleagues to test the tool in low-resource settings (Pg. 14, par. 3).*
